# Use of email, cell phone and text message between patients and primary-care physicians: cross-sectional study in a French-speaking part of Switzerland

**DOI:** 10.1186/s12913-016-1776-9

**Published:** 2016-10-05

**Authors:** Jonathan Dash, Dagmar M. Haller, Johanna Sommer, Noelle Junod Perron

**Affiliations:** 1 University of Geneva Faculty of Medicine, Geneva, Switzerland; 2 Unit of Primary Care Medicine, University of Geneva Faculty of Medicine, Geneva, Switzerland; 3 Division of Primary Care, Department of Community Care, Primary Care and Emergency, Geneva University Hospitals, Geneva, Switzerland; 4 Unit of Development and Research in Medical Education, University of Geneva Faculty of Medicine, Geneva, Switzerland

**Keywords:** Email, Cell phone, Text message, Communication, Physician, Patient, Primary care

## Abstract

**Background:**

Physicians’ daily work is increasingly affected by the use of emails, text messages and cell phone calls with their patients. The aim of this study was to describe their use between primary-care physicians and patients in a French-speaking part of Switzerland.

**Methods:**

A cross-sectional mail survey was conducted among all primary-care physicians of Geneva canton (*n* = 636). The questionnaire focused on the frequency of giving access to, type of use, advantages and disadvantages of email, cell phone calls and text messages communication between physicians and patients.

**Results:**

Six hundred thirty-six questionnaires were mailed, 412 (65 %) were returned and 372 (58 %) could be analysed (37 refusals and three blanks). Seventy-two percent physicians gave their email-address and 74 % their cell phone number to their patients. Emails were used to respond to patients’ questions (82 %) and change appointments (72 %) while cell phone calls and text messages were used to follow patients’ health conditions. Sixty-four percent of those who used email communication never discussed the rules for email exchanges, and 54 % did not address confidentiality issues with their patients. Most commonly identified advantages of emails, cell phone calls and text messages were improved relationship with the patient, saving time (for emails) and improving the follow-up (for cell phone and text messages). The main disadvantages included misuse by the patient, interference with private life and lack of reimbursement.

**Conclusions:**

These tools are widely used by primary-care physicians with their patients. More attention should be paid to confidentiality, documentation and reimbursement when using email communication in order to optimize its use.

**Electronic supplementary material:**

The online version of this article (doi:10.1186/s12913-016-1776-9) contains supplementary material, which is available to authorized users.

## Background

Electronic communication dominates our current world. More than 3.3 billion e-mail accounts were opened in 2012 and this figure will reach 4.3 billion in 2016; every day 144.8 billion emails are sent in the world [1]. In Switzerland in 2014, more than 91 % house-holds had access to internet and nearly 75 % of the population above the age of fourteen used Internet every day. Similarly, more than half of the world population owns a cell phone [2]. In Europe, several countries have a mobile phone-population ratio higher than one; in Switerland, the ratio was 1.4 in 2014 [3].

Use of electronic communication in healthcare is driven by the idea it can improve accessibility, quality and performance of health service delivery [4]. More than 85 % of physicians use the internet and 55 to 64 % use email to correspond with colleagues and support staff [5]. Ten years ago, 20–25 % of European general practitioners reported using email to communicate with their patients [6]. Increasingly patients are willing to use electronic media to communicate with their physician. It provides them with a feeling of safety and easier access to care. Yet a significant percentage of physicians are reluctant to exchange emails with their patients [7, 8].

The use of cell-phone communication in health care has recently been subject to less studies but its use does not seem to have decreased with the rise of email communication [4, 9]. In a study published in 2011, about half of the physicians offered to communicate with their patients by telephone [10]: it allows them to choose to answer according to specific time slots, disconnect the phone when needed and avoid miscommunication [10–12]. Text message is commonly used in health care settings. However, a systematic review on the use of text messages in health care indicates that it is used mostly for one-way communication from providers to patients, disease management, administrative processes or preventive care [13].

Most studies that have addressed the use of emails and cell phone communication in health-care settings were conducted in the early 2000’ and mainly in the United States and Israel. Use of email communication in Europe appears to be extremely variable and closely linked to a country’s main health policy (development of ehealth): the percentage of citizens (aged 16–74 years old and internet users) who reported sending or receiving en email form their doctor-nurse or health care organization varied from 18.7 % (France) to 50.7 % (Denmark) [14]. Little is known on the use of such communication tools in Switzerland. The aim of this study was to explore the frequency and type of use of email, cell phone and text message communication by primary-care physicians in a French-speaking part of Switzerland. We were also interested in primary-care physicians’ views about the advantages and disadvantages of using these communication tools. Finally we sought to explore whether physicians established rules for use of email to communicate with patients.

## Methods

### Design, context and participants

We conducted a cross-sectional study among primary-care physicians working in Geneva, Switzerland, which is a multicultural urban canton of 470,000 inhabitants (40.2 % foreigners). In the Swiss health system, primary-care physicians include general internists, paediatricians and medical practitioners (practitioners with at least 3 years of postgraduate training).

We invited all primary care physicians currently practicing in Geneva to participate in this survey (*n* = 636). The list was provided by the Geneva Medical Association.

### Questionnaire

Following a literature review [8, 10, 15–17], we developed a 33 item questionnaire (Additional file 1). The frequency of giving access, topics addressed, advantages and disadvantages were explored for email, cell phone and text message communication through multiple choice questions. Questions about variety of uses, reimbursement and confidentiality issues were collected only for email communication.

The questionnaire was pretested with three primary-care physicians for clarity and comprehension purposes and was subsequently modified and improved. The questionnaire was sent by post in August 2013 to all primary-care physicians, followed by a reminder 6 weeks later.

The study was granted a waiver from approval by the Ethical Committee of the Canton of Geneva since it did not involve collecting any personal health information (article 2 of the Swiss Federal Act on Research involving Human Beings) [18]. Participants were informed that the data would be analysed and reported once anonymised.

### Analysis

Stata software version 12.0 was used for the analysis. Participants’ responses, regarding their use of email, cell phone calls and text messages were analysed descriptively using percentages (Additional files 2 and 3). We conducted multivariate analyses using logistic regression to identify physician characteristics (gender, age-group, % activity, practice location, solo versus group practice) associated with the use of email and/or cell phone communication.

## Results

Of 636 questionnaires that were sent, 412 (65 %) were returned. Excluding uncompleted questionnaires (37 refusals and three blanks), 372 (58 %) questionnaires were available for analysis regarding e-mail communication and, because some participants did not respond to questions situated at the back of the questionnaire sheet, 322 (51 %) were available for the analysis in relation to the use of cell phone and text message communication.

Table 1 shows that there was an equal number of male and female participants; most of them were over 49 years old and worked in an urban area. Eighty-seven percent of participants (*n* = 324) used the computer for billing purposes and 48 % (*n* = 179) used it to write prescriptions, 44 % (*n* = 163) to enter patient information in the electronic medical file and to show results to patients.

**Table 1:** Participants’ sociodemographic characteristics (*N* = 372)

Characteristics	*n* (%)
Gender:	
Female	173 (46.6)
Age (yrs old):	
0–39	43 (12.9)
40–49	96 (27.0)
50–65	182 (52.3)
> 65	27 (7.8)
Full time equivalent:	
< 0.25	4 (1.1)
0.25–0.50	27 (7.5)
0.51–0.75	80 (22.1)
> 0.75	251 (69.3)
Type of practice:	
Solo	150 (42.6)
Groupe	186 (52.8)
University affiliated	15 (4.3)
Other	1 (0.3)
Practice location:	
Urban	276 (76.5)
Suburban	80 (22.2)
Rural	5 (1.4)
Postgraduate training level:	
Still in training	2 (0.6)
Practicing physician	22 (9.1)
Specialist title in general internal medicine	211 (58.3)
Specialist title in paediatrics	71 (19.6)
Additional certified training	35 (9.7)
Other	10 (2.8)

### Providing access to, topics, advantages and disadvantages of email, cellphone and text message communication

Seventy two percent of participants (266 out of 372) reported offering the possibility to communicate by email and 70 % (226 out of 322) gave their cell phone number to their patients. However, most physicians only provided such opportunity to a minority of their patients (Table 2) and reported using email and cell phone or text message only 1 to 5 times a month. Regarding emails, 61 % participants reported managing email communication personally, 25 % shared email management with their secretary and 12.4 % had two different professional emails, one for medical information and one for the office management (appointments, schedules).

**Table 2:** Frequency of use, topics, advantages and disadvantages of email, cell phone and text message communication reported by participants

	Email (*n* = 266)	Cell phone number (*n* = 226)	Text message (*N* = 226)
	*n* (%)	*n* (%)	*n* (%)
Giving access to:			
1–25 %	163 (61)	163 (72.1)	Giving access to cell phone number includes text messages.
25–50 %	21 (7.9)	15 (6.6)	
50–75 %	3 (1.1)	7 (3.1)	
> 75 %	7 (2.6)	41 (18.1)	
All (appointment e-mail address indicated on the appointment card)	72 (27.0)	NA	
Use initiated by:			
Physician	60 (22.8)	155 (69.2)	79 (47.6)
Patient	101 (38.4)	22 (9.8)	35 (21.1)
Both	103 (38.8)	47 (21.0)	52 (31.3)
Topics:			
Change of appointment	192 (71.9)	55 (24.3)	60 (26.5)
Test results	71 (26.6)	138 (61.1)	77 (34.1)
Follow-up of patient’s health	132 (49.4)	187 (82.7)	107 (47.3)
Patients’ questions	219 (82.0)	124 (54.9)	88 (38.9)
Other^a^	39 (14.7 %)	25 (11.1)	14 (6.2)
Advantages:			
Time saving	119 (44.6)	94 (41.6)	94 (41.6)
Less consultations	104 (39.0)	105 (46.5)	50 (22.1)
Improved follow-up	95 (35.6)	148 (65.5)	81 (35.8)
Improved relationship	129 (48.3)	142 (62.8)	85 (37.6)
Other^b^	41 (15.4 %)	25 (11.1)	22 (9.7)
Disadvantages:			
Misuse	86 (32.2)	89 (39.4)	56 (24.8)
Encroachment on private life	85 (31.8)	131 (58.0)	90 (39.8)
Misunderstanding	44 (16.5)	27 (11.9)	35 (15.5)
Waste of time	61 (22.8)	27 (11.9)	23 (10.2)
No billing	81 (30.3)	54 (23.9)	45 (19.9)
Other^c^	35 (13.2 %)	21 (9.3)	15 (6.6)

^a^Other: medication prescription, provision of information

^b^Less anxiety, no advantages

^c^No disadvantages, impatience

The multivariate analysis taking into account physicians’ gender, age-group, practice location, work percentage, and type of practice showed that there were no gender differences but that older doctors were less inclined to provide their email address to patients (OR 0.29, 95 % CI: 0.87–0.94, ﻿*p*﻿ 0.027; and 0.25, 95 % CI 0.71–0.89, ﻿﻿*p*=0.033, respectively for the age group 50–65 and > 65 years, compared to those <40 years old). Similarly younger physicians and those working in an urban context were more likely to give their email address to more than 75 % of their patients, though the latter relationship just failed to reach statistical significance (0R 1.93, CI 0.99–3.78, *p* = 0.054). None of the physicians’ characteristics were associated with their use of the cell phone or text messages to communicate with patients.

Cell phone calls were more often initiated by physicians than email or text messages (Table 2). The main topics reported by physicians for email exchange were answering patients’ questions and changing appointments. Cell phone calls were used essentially to follow-up on patients’ health and communicate test results while text messages were used equally for all the mentioned topics (Table 2).

Email exchanges helped them save time and improved their relationship to patients while improved follow-up and relationship with patients were the main benefits associated with the use of cell phone communication (Table 2). Time saving was the main advantage associated with using text messages.

Disadvantages for using email, cell phone and text message communication included misuse and encroaching on private life (Table 2). In addition, waste of time and lack of reimbursement were more often mentioned for email communication.

### Reasons and types of use for email communication

Participants reported using email communication essentially to reassure their patients, improve the relationship and avoid unnecessary consultations (Table 3). A vast majority of participants reported that they did not discuss confidentiality issues with their patients and did not negotiate rules about the number and/or content of emails or the delay to answer. Half of the participants reported that they answered emails at any time and only a minority did not answer emails on weekends or evenings. A large majority of them did not report charging email communication and only a minority of them documented email communication in patients’ medical files. In the multivariate analysis, working in a solo practice was significantly associated with a lower tendency to systematically charge email communications (OR 0.37 95 % CI 0.16–0.87, *p* = 0.022).

**Table 3:** Participants’ reasons and types of use of email communication

Reasons and types of uses	*n* (%)
Reasons for using email communication:	
To reassure the patient	130 (48.7)
To reassure the doctor	29 (10.9)
To improve the patient-doctor relationship	106 (39.7)
To save time	57 (21.3)
To avoid unnecessary consultations	119 (44.6)
Other^a^	54 (20.2)
Discussion of confidentiality issues:	
Never	144 (53.9)
1–25 %	30 (11.2)
25–50 %	13 (4.9)
50–75 %	6 (2.2)
Always	59 (22.1)
Discussion of rules of use:	
No	170 (63.7)
Number of emails	22 (8.2)
Content of emails	34 (12.7)
Delay to answer	55 (20.6)
Time of answer:	
Any time	138 (53.7)
During the week but not on weekends	60 (23.3)
Never in the evenings or on weekends	28 (10.9)
Specific slots	18 (7.0)
Other	13 (5.1)
Reimbursement of email communication:	
Never	162 (63.0)
1–25 %	38 (14.8)
25–50 %	19 (7.4)
50–75 %	18 (7.0)
Always	20 (7.8)
Documentation of email communication in the medical file:	
Never	27 (10.4)
Rarely	26 (10.0)
Occasionally	46 (17.8)
Often	66 (25.5)
Always	94 (36.3)

^a^Travelling patient, chronic disease follow-up

## Discussion

This study shows that nearly 72 % primary-care physicians in Geneva Switzerland gave their patients access to email communication and 70 % gave their cell phone number to their patients in 2013. Physicians felt that all communication tools helped them build a better relationship with their patients as well as gain time but were associated with the risk of misuse and encroachment on private life. Doctors uncommonly discussed confidentiality issues in relation to email communication with their patients, and more than half of them did not bill such communication.

The rate of physicians offering email communication to their patients is higher than what has been previously reported and naturally follows the constant rise of email use in everyday life [19]. However, primary care physicians still remain cautious in its use and restrict it to a minority of patients [20]. The fact that physicians use email, phone or text message communication for different purposes suggests that these tools respond to different needs. Sophisticated web based health systems providing online communication (called patient portal), as implemented in many U.S. and U.K. healthcare institutions, may therefore not replace phone-based interactions [21–23]. For example, phone communication is still considered by both physicians and patients as a way to improve care and follow-up and to reduce unnecessary visits to emergency services [4, 10, 11]. Patients also expect physicians to phone to communicate test results–it is thought to improve patient understanding, provide reassurance and increase quality and continuity of care [24, 25].

However, the reported use of email communication for changing appointments suggests that most practices do not have effective incoming email triage processes. Several studies show that appropriate triage of incoming messages by office staff or web-based messaging systems helps manage email flows, improve the efficiency of office communications with patients and increase patient safety and physicians’ acceptance of email communication with patients [26].

The disadvantages of email and phone communication relate to disruption of physicians’ privacy, lack and waste of time and poor or misuse of communication [10, 16, 27, 28]. Two factors may explain such negative perceptions. First, most physicians did not report establishing rules of use. As in many countries, email communication still occurs in an unstructured way [29] and do not follow common rules such as setting limits by using system templates, limiting free text to 2–3 sentences, defining time limits and time slots to answer or systems to cover physicians’ absences [26]. Second, although patients may not be opposed to physicians billing the time spent for an email [8], most Swiss physicians do not bill email communication. Lack or inadequate compensation is a commonly reported disadvantage and is seen as a barrier to physician’s adoption of email communication [10, 17, 30].

Despite the fact that several recommendations about confidentiality have been formulated over the last 10 years [19, 31, 32], the percentage of primary care physicians in our study who did not discussing confidentiality issues in relation to email communication with their patients is similar to that reported in studies conducted in other countries 5 to 10 years earlier (36 to 60 %) [15, 16, 33]. Until quite recently, use of secured email communication was essentially reported in large health maintenance organisations [34, 35]. In Switzerland, HIN was set up to ensure the safety of email communication between health practitioners but not between physicians and patients [36]. The ongoing development of ehealth environments including administrative and messaging capabilities in addition to medical record access will definitely improve the safety of electronic communication beside [26]. However, adoption of such environments in solo or small primary care practices may take time. In the meantime, we believe that it is the role of the national health organisation systems in charge of medical regulations to acknowledge and value this type of clinical communication and set ethical and compensation guidelines in line with expected standards of care [9].

The study has several limitations. The response rate (64 %) was not optimal and a number of participants (13 %) did not complete the questions on cell phone and text message use, preventing the generalisability of our findings to all Geneva primary-care doctors. In comparison to previous surveys of doctors, our response rate, however, was high [37]. We focused on the doctors working in an urban area and it is possible that profiles of use of these different modes of communication differ in less dense areas of Switzerland or elsewhere. We did not collect data on documentation and billing of cell phone and text message communication since we did not want the questionnaire to be too long in order to favour a high response rate. Yet clinical experience suggests that these might be even lower than for email use. The questionnaire did not enable us to clearly differentiate practice patterns and perceptions between physicians handling a single, a shared or two different e-mail addresses. Finally, we did not explore the use of social media (e.g. Facebook) as a communication tool between physicians and patients. As the mean age of practicing physicians in Geneva is over 50 years, use of social media in this population is still likely to be uncommon. Health system websites, currently offered in some countries, are still in development and have not yet been implemented on a large scale in Switzerland.

## Conclusions

This study shows that email, text message and cell phone are widely used between an urban population of primary-care doctors and their patients in Switzerland. Since electronic communication in healthcare is meant to expand with the implementation of eHealth environments in Switzerland and the collection and use of patient-generated health information [38], federal health organisations are urgently invited to establish recommendations of good use and rules for confidentiality and reimbursement. This would optimize the use of this computer-based technology and help integrate one of the ten “building blocks for a performing primary care” into the Swiss health system [39].

## Additional files

Additional file 1: Questionnaire - email and SMS survey. (XLSX 12 kb)
Additional file 2: List of codes - email and SMS survey. (DOCX 203 kb)
Additional file 3: Database - email and SMS survey. (CSV 84 kb)

## Abbreviations

UK: United Kingdom
US: United States
