# Supplementary material for: Use of email, cell phone and text message between patients and primary-care physicians: cross-sectional study in a French-speaking part of Switzerland
Source: BMC Health Serv Res. 2016 Oct 5;16:549. doi: 10.1186/s12913-016-1776-9 (PMC5051025; doi:10.1186/s12913-016-1776-9)
Supplement: Additional file 2: — List of codes - email and SMS survey. (DOCX 203 kb) [file 12913_2016_1776_MOESM2_ESM.docx]

Geneva, 25 September 2013

**Concerns : Use of email and mobile phone**

Madam, Sir,

As a 4th year medical student, I chose to undertake my Master’s degree project on a subject concerning family medicine, under the supervision of Dr.Junod Perron of the Division of primary care of Geneva University Hospitals, in collaboration with the primary care unit of the faculty of medicine of Geneva University.

I would be very grateful if you could take a few minutes (max 10 minutes) to answer the attached questionnaire. It concerns the use of email, mobile phone and SMS between family doctors and patients (or parents to paediatricians) outside the consultation.

The questionnaire must be completed by the person whose name is on the envelope. It has an identification number so that I avoid sending reminders to those who have already answered. Please answer all the questions by checking the box corresponding to the selected answer. If you do not know what to answer, choose the closest answer to your opinion or situation. Your comments are most welcome

After completing the questionnaire, please put it in the enclosed envelope and send it back. If you are in Switzerland, there is no postage stamp required. All information collected in this survey will be treated confidentially and the results presented in an anonymized way. Your answers will never be connected with your name.

Thank you in advance for your participation,


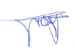

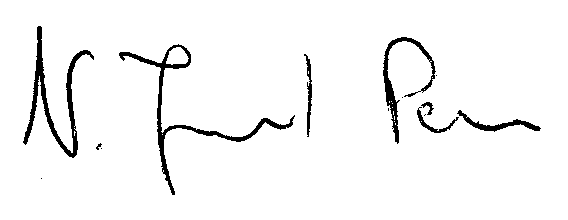

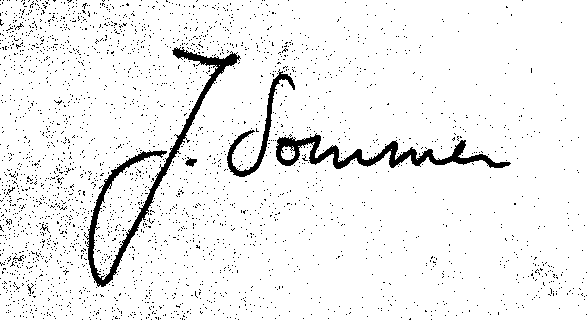


Jonathan Dash Noëlle Junod Perron Johanna Sommer


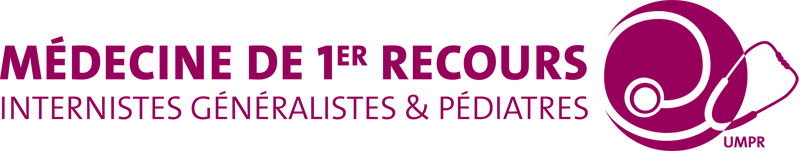


| NB : Return your questionnaire **empty** if : | - You do not wish to participate to this survey |  |
| --- | --- | --- |
|  | - If you are no longer working |  |
|  |  |  |

For more information, please contact me:
076 616 06 26

If you have lost the envelope, please return the questionnaire to the following address:

**Enquête « email », Dr. N. Junod Perron, Service de médecine de premier recours, Département de médecine communautaire, de premier recours et des urgences-HUG, 4 rue**

**Gabrielle Perret-Gentil, CH-1211 Genève 14**

**Questionnaire concerning doctor-patient communication by e-mail**

**1. Your age: ……..**

(please tick the appropriate box )

**2. You are ?**

A male………………………………………………… 1

A female…………………………………………….... 2

**3. The percentage of your working time is of:**

<25%…………………………………………………….. 1

Between 25% and 50%.............................................. 2

Between 50% and 75%....................................……... 3

>75%................................................................……... 4

**4. You work in a:**

Private practice in solo...………………………………. 1

Private practice in a group...............................…....... 2

Ambulatory University Hospital…................................ 3

Other :

**5. Your place of work is in an area:**

Urban……..……………………………………………... 1

Semi urban..………………………....................……… 2

Rural..….………………………………………………… 3

Comments :

**6. You practice as that of a:**

Internist in training…………...…………………………...... 1

Medical practitioner……..………………………............... 2

FMH (Swiss specialist title) internal/general medicine.……………………... 3

FMH paediatrician………..………………………………... 4

In further training………….…...…………………………… 5

If yes, which one…………………………………………

Other…………………………………………………………  6

Comments :

**7. For what purpose do you use computer?**

*(Several responses possible)*

For billing ……………………………………..................... 1

For medical prescription ……….…………………………. 2

To enter data of consultations ……................................ 3

To show the patients the results/ diagrams ………....... 4

To have access to external information …………………. 5

other : ………………………………..……………............. 6

Comments:

**8. Do you give your e-mail address to your patients?**

*(If no, go to the question 22)*

No….………………………………...…………………….. 1

Yes, 1-25% of my patients have my e-mail.………… 2

Yes, 25-50% of my patients have my e-mail ….......... 3

Yes, 50-75% of my patients have my e-mail …….….. 4

Yes, >75% of my patients have my e-mail ……..…… 5

Yes, all of my patients as this information is on my

card ……………………………………………………… 6

Comments :

**9. During the last month, how many e-mails have you exchanged with your patients?** …..

**10. With how many patients have you exchanged e-mails:**

Regularly: ……………./month

Sporadically: ……………./month

Comments :

**11. Who initiated the e-mail address exchange?**

It is you who has proposed most of the time ………… 1

The patients have asked for it most of the time ………………………………………………………… 2

Both you and the patients ……………………………………………………………… 3

**12. Who has access and who handles the reception of e-mails?**

You are the only one who has the access hence it is you alone who handle the e-mails…………………….. 1

Your secretary (medical) has access also, but you alone handle the e-mails…..……………………………… 2

Your secretary sorts out the e-mails before you receive them …………………………………………………..…………… 3

You have a practice address to contact the secretary and a private address for medical information …...… . 4

Other : ...…………………………………………………. 5

Comments :

**13. From the exchange of e-mails which you have from your patients, what are the subjects mostly dealt with?** *(Several responses possible)*

Changing of appointment …………………..…………… 1

Investigation results……………………..……………….. 2

Follow-up of patient’s health …………….…….............. 3

Queries from patients ……………………………… 4

Other : …………………………………………............... 5

Comments :

**14. For what good reasons do you give your e-mail address to your patients?**

*(Several responses possible)*

To reassure them…………...…………………………….. 1

To reassure yourself…………..…………………………... 2

To improve doctor-patient relationship ……..…………… 3

To lose less time in consultation ……………..………... 4

To avoid any extra consultation ……………….………. 5

Other :………………………………………….………… . 6

Comments :

**15. Among what percentage of your patients do you discuss the rules of confidentiality of e-mails?**

With 100% of my patients …………………………... 1

~ 75% of my patients …........................................... 2

~ 50% of my patients......................................……... 3

~ 25% of my patients......................................……... 4 With none of my patients.................................…….........5

Comments :

**16. When do you respond to e-mails?**

Whenever including evening and week-ends …….….. 1

During the week but not at week-ends ………………... 2

Neither in the evening nor at week-ends……….….. 3

At specific timeslot…………….………………………... 4

Other.. : ....................................................................... 5

Comments :

**17. What percentage of e-mails do you charge?**

I do not charge for my e-mails…………………………... 1

<25% of the e-mails …………………………………….. 2

25 to 50% of the e-mails………………………………… 3

between 50 and 75% of the e-mails…………………….. 4

I charge for every e-mail …………………………………. 5

Comments :

**18. What advantages do you get from the exchange of e-mails with your patients?** *(Several responses possible)*

Save time ………………..…………………………. 1

Less unnecessary consultation …………………….. 2

Better follow-up ……………………....……………………. 3

Better relationship with the patient ……………………… 4

Other :……………………………………………………. 5

Comments :

**19. What are the main disadvantages that you have to face?** *(Several responses possible)*

Misuse of the e-mail …………………………….………... 1

Interference in private life………………………………. 2

Misunderstanding ……….……………….……............. 3

Waste of time ………………………………………….. 4

None remuneration ………………………………………. 5

Others:.. …………………………………………............. 6

Comments :

**20. Do you keep a copy/ document of the exchange of e-mails in the medical file?** *(Several responses possible)*

Always…………………………………………………...... 1

Often…..………………………………………...………… 2

Sometimes………………………………………..….......... 3

Rarely……………………………………….…….….. 4

Never………………………………………...……..…… 5

Comments :

**21.** **Do you discuss the guidelines, which need to be respected when you exchange your e-mail addresses?** *(Several responses possible)*

Yes, above all regarding the number of e-mails………….. 1

Yes, above all regarding the content of the e-mails… 2

Yes, concerning the time of the response ………………… 3

No……………………………………………………...…. 4

Comments :

**22.** . **If you have answered no to the question 8, why do you not give your mail address?** *(Several responses possible)*

**Please turn the page**

**Questionnaire on telephone conversations**

**23. Do you give your mobile telephone number to your patients?**

No.………………………………………………………. 1

Yes, 1-25% of my patients have my number…....... 2

Yes, 25-50% of my patients have my number……... 3

Yes, 50-75% of my patients have my number……... 4

Yes, > 75% of my patients have my number…......... 5

**24. During this last month how many telephone calls have you exchanged with your patients?** .....

**25. Who initiated the exchange of the mobile phone numbers?**

You have proposed that most of the time…………….. 1

The patients have asked for it most of the time……………………………………………................ 2

You as well as the patients………………..….…………. 3

Comments :

**26. During your telephone conversation with your patients, what are the subjects that are discussed?** *(Several responses possible)*

Changing of appointments…………………………. 1

Investigation results…..……………………….......... 2

Follow up of patient’s health conditions………………. 3

Queries from patients…………… ………………. 4

Other : ...……………………………………………….. 5

Comments :

**27. What are the main advantages of telephone conversations with your patients?** *(Several responses possible)*

Saving time…………………………………………... 1

Less unnecessary consultations…………..……….. 2

Better follow-up………………………….…………........... 3

Better relationship with the patient……………….......... 4

Other: …………………………………………............. 5

Comments :

**28. What are the main disadvantages that you have to face?** *(Several responses possible)*

Misuse of utilization of telephone…………………….. 1

Interference with private life ………………………… 2

Misunderstanding …….……………………….......... 3

Waste of time ………………………………………….. 4

None remuneration ………………………………………. 5

Other :………………………………………….……….. 6

Comments :

**Questionnaire on SMS exchange**

**29** **During this last month, how many SMS have you exchanged with your patients?** …..

**30. Who proposed to use SMS ? :**

It is you who proposed it most of the time ………. . 1

The patients have asked for it most of the time ………. 2

You as well as the patients ……………………………. 3

Comments :

**31. During your exchange of SMS with your patients what are the subjects that are discussed?** *(Several responses possible)*

Changing of appointments …………………………… 1

Investigation results ………………………............. 2

Follow up of patient’s health conditions …………………. 3

Queries of patients ………………… …………… 4

Other : ...…………………………………………………. 5

Commentaires :

**32. What are the main advantages of the exchange of SMS with your patients?** *(Several responses possible)*

Saving time …………………………………………….. 1

Less unnecessary consultations …..………………. 2

Better follow-up ……………………… ………….............. 3

Better relationship with the patient ………………............. 4

Other: …………………………………………............... 5

Comments :

**33. What are the main disadvantages that you have to face?** *(Several responses possible)*

Misuse of utilization of telephone…………………….. 1

Interference with private life ………………………… 2

Misunderstanding …….……………………….......... 3

Waste of time ………………………………………….. 4

None remuneration ………………………………………. 5

Other:………………………………………….……….. 6

Comments :

THANK YOU FOR ANSWERING
